# On the Minimum Quantity of Mobile Sensor Nodes for Full Coverage in Hybrid WSN

**DOI:** 10.3390/s25103210

**Published:** 2025-05-20

**Authors:** Monik Silva Sousa, João Viana da Fonseca Neto

**Affiliations:** 1 Laboratory of Embedded Systems and Intelligent Control, Federal University of Maranhão-UFMA, São Luís 65080-805, Brazil; 2 Department of Electrical Engineering, Federal University of Maranhão-UFMA, São Luís 65080-805, Brazil; joao.fonseca@ufma.br

**Keywords:** circular formation, coverage hole, full coverage, hybrid WSN, sensor nodes quantity

## Abstract

A main challenge in deploying wireless sensor networks (WSNs) is determining the minimum quantity of sensor nodes required to fully cover the region of interest while avoiding coverage holes. This study proposes a method to compute the number of nodes needed to monitor a circular region and a distributed control strategy based on circular formations to move dynamic agents to their desired positions. The method addresses the coverage problem, ensuring that each point in the monitored region is detected without losing connectivity. In addition, the study compares this approach with a sensor node allocation method based on Voronoi diagrams, highlighting the need for an algorithm that computes the desired positions of the agents to provide guaranteed flawless coverage; the proposed method achieves this by obtaining the desired final positions. The hybrid WSN architecture, together with the proposed method, achieves full coverage efficiently and better utilizes the detection circumference of sensors compared to traditional rectangular monitoring regions.

## 1. Introduction

Technological innovations in the areas of wireless communications, digital electronics, and microprocessors have enabled a revolution in remote sensing, stimulating studies and research that have led to the creation of wireless sensor networks (WSNs). These networks can be applied to the monitoring, tracking, coordinating, and processing of hostile environments where human intervention is impossible or difficult to access, such as areas related to chemical, biological, and physical processes in aquatic environments, among others.

A WSN is a group of sensors collaborating with each other to achieve a specific monitoring objective, where the network already overcomes the limitation of a single sensor in detecting a small region. These sensors collect data and use them for decision-making together with data from their adjacent neighboring sensors [1]. However, these WSNs suffer from some restrictions, such as the sensor’s detection range, battery use, connection capacity, memory, and computing capabilities. This study discusses two of these restrictions—namely, detection radius and connectivity—taking into account the fact that these are factors that impact network coverage. Another factor that directly impacts coverage is residual energy. However, this work focuses on determining the minimum quantity of mobile sensor nodes needed to ensure full coverage of a given circular monitoring region.

The sensor detection model is also a fundamental requirement for the coverage of the monitoring region, influencing network performance. The traditional models include the binary disk model [2,3] and the directed angle model [4,5,6]. In this paper, the binary disk model is selected, in which a circumference determines the sensor’s sensitivity, and the points within it are fully covered, assuming that there is no obstacle in the monitored environment and ignoring the decrease in the detection signal; that is, it considers that this signal is constant within the sensor’s detection circle [1]. This binary disk model was chosen for its flexibility, where each sensor is characterized by a circular detection area, with the sensor positioned at the center and its range defined by a radius. As a result, any target within this disk is reliably detected, simplifying coverage planning while ensuring high efficiency in applying coverage across various geometries.

The sensor nodes that make up a WSN can be static or dynamic. These sensors define the network’s classification, determining whether it will be static, dynamic, or hybrid. Hybrid WSNs are the most suitable for hostile or uncontrolled environments, as mobile nodes provide adaptability to the network, filling coverage holes and optimizing network lifetime [7].

The network topology used is the hybrid star–ring, which has attributes of both the star and ring topologies. This model takes advantage of the low energy consumption present in the star topology compared to other network topologies. However, its reliability is low due to its dependence on a single node to manage the entire network. Therefore, we also leverage the simplicity of the ring topology, in which the sensor nodes are connected through a circle, and each node communicates with its two adjacent neighbors to increase the network’s reliability [8].

In a WSN, coverage is one of the fundamental requirements to guarantee quality of service (QoS), which is thus considered a WSN performance metric [9], as it indicates how well each point in the detection field is covered [8]. Consequently, to ensure full and secure coverage, it is necessary to determine the type of coverage and design a strategy for allocating sensor nodes in such a way that the least number of nodes are used while ensuring a high degree of coverage of the region [10]. An alternative is to use unmanned aerial vehicles as mobile nodes to achieve optimal coverage efficiency [11].

WSNs are widely used to monitor hostile environments, such as aquatic environments, and consist of intelligent sensor nodes capable of detecting and classifying pollutants [12]. The quantity of sensor nodes is critical because it is directly related to energy consumption and the redundancy of measured data. Therefore, Afizudeen et al. [13] proposed a deterministic sensor node deployment algorithm based on the Grundy number, with the aim of using an efficient quantity of sensor nodes, maximizing WSN lifetime, and minimizing the redundancy of acquired data.

With regard to determining the minimum quantity of sensor nodes to ensure complete coverage, Chunyang et al. [14] proposed a new coverage scheme using a hybrid sensor network, which aimed to fully cover a rectangular monitoring region using a minimum quantity of mobile sensor nodes, thus reducing the network’s energy consumption and increasing its lifetime. The minimum quantity of sensor nodes to cover the rectangular area was computed mathematically. Then, the minimum quantity of mobile sensor nodes was computed in order to balance energy consumption and the coverage of the WSN. An algorithm called virtual force, as well as Voronoi graphs, were also proposed to improve coverage and reduce the movement distance of mobile nodes. Another topology construction, as well as maintenance based on a trust algorithm, was used to further extend the WSN lifetime. The researchers were able to prove that the proposed scheme achieved a maximum coverage of almost 100% using a minimum number of mobile nodes, proving its efficiency.

WSN coverage is a current research field that has shown some relevant progress. However, to ensure efficient coverage, mobile agents must move in a coordinated manner to their specified positions. This movement can be defined using different methods currently available in the literature, such as multi-agent formation, optimized approaches, and virtual force.

In [6], the authors investigated closed barrier coverage using a hybrid directional sensor network. The monitoring area considered is rectangular, with sensors initially distributed randomly, with some of them mobile and some static. Subsequently, these sensors were selected and arranged to form the coverage barrier. The minimum quantity of mobile sensors required was determined based on the computation of the gaps between the static sensors, taking into account the angle of view and the detection radius of the sensors, together with geometric information of the area. In addition, the authors proposed a strategy for rotating and moving the sensors, aiming to achieve closed barrier coverage with the smallest possible quantity of mobile sensors and optimizing energy consumption. The experimental results confirmed the effectiveness of the proposed approach.

Wang et al. [15] studied the circular formation of dynamic agents with order preservation along the entire trajectory of the sensors such that no collision occurred during circular formation. In another study by Wang et al. [16], the measurement error was added in relation to the position that a sensor detects from its adjacent neighbors. This proved that the proposed distributed control law leads the system to the desired formation, even with noisy measurements regarding the agents’ positions. Another method to avoid collision in a multi-agent system was studied in [17], in which a method based on the effects of centralization and decentralization for cooperative control was proposed, utilizing an anti-Laplacian matrix to control the agents when unmanned aerial vehicles are on conflicting routes.

In the same line of research, Feng et al. [18] worked on formation control for mobile sensor networks with limited position measurement errors, in which the sensor nodes were required to perform a determined formation in a circle. They solved this problem in two stages: in the first stage, an estimation algorithm was proposed to estimate the position difference between the sensors and their neighbors. In the second stage, they proposed a distributed circle formation control law for each sensor, in which they used the estimated position difference as data, thus showing that the effect of the measurement error in circle formation is reduced.

Regarding the circle formation problem, Shen et al. [19] worked on the problem of controlling the formation of a circle in finite time for a network of mobile agents with continuous time dynamics, in which distributed control laws were derived and led the agents to the prescribed circle formation. The theoretical analysis of this proposed method was carried out using the finite-time stability theory and Lyapunov methods. The research developed by Wu et al. [20] described an important point for WSNs: connectivity. They talk about the control of the formation of a circle for a multi-agent system with preservation of connectivity, in which a formation control law is proposed for the preservation of network connectivity and the saturation of the input signal, maintaining connectivity during formation.

Song et al. [21] considered the optimal implementation of mobile agents with different velocities when they are limited to moving in a circle; the main objective was to achieve the final configuration of the multi-agent network and minimize the agents’ arrival time at any point on the displacement circumference.

Runliang et al. [22] proposed a full coverage method, ensuring that each monitoring point was covered by at least one sensor node, in addition to providing energy efficiency to the network based on an improved Gray Wolf algorithm. Kumari et al. [23] proposed the development of an algorithm based on mixed integer linear programming, with the objective of first allocating the set of static nodes and then implementing the set of mobile nodes in order to maximize the coverage area and minimize the amount of node movement to achieve the desired coverage.

Regarding the positioning of sensor nodes, Kumar et al. [24] proposed an optimized approach for node positioning in an underwater acoustic network, which aimed to maximize the coverage area of the network and increase its lifetime. To achieve this goal, they used two techniques: Voronoi-fuzzy C-means, in which the Voronoi technique is used to divide the coverage area into distinct regions; the fuzzy C-means algorithm is employed to classify the regions and position the nodes more efficiently, improving the distribution of the nodes and avoiding excessive overlap. The other technique is salp swarm optimization, used to optimize the positioning of nodes over time, dynamically adjusting the positions of the nodes to maximize the coverage area and, at the same time, minimize energy consumption; this algorithm is inspired by the behavior of salps.

The works cited above demonstrate the importance of determining network topology, the number of sensor nodes, and coverage control needed to obtain efficient, continuous, and reliable monitoring. Consequently, this study aims to propose a dynamic form of coverage without holes, constituting dynamic and static sensor nodes; the quantity of sensor nodes needed to monitor a region without holes and without loss of sensor connectivity is determined according to the sensor detection radius, the connectivity radius, and the region to be monitored, as a result providing the number of agents and the position of the mobile sensor nodes.

In scenarios characterized by limited accessibility or adverse environmental conditions, the proposed methodology has proven to be effective in enabling reliable monitoring. It directly contributes to the preservation of ecosystems, mitigating the negative impacts of human activity while promoting innovation in the area of WSNs. Ensuring the minimum quantity of mobile sensor nodes required for coverage without holes reduces operational costs associated with idle or redundant sensors.

The network configuration adopted in this work follows a star–ring topology composed of a static central sensor node and multiple mobile sensor nodes arranged in a circular formation. This architecture increases the flexibility and adaptability of the network, qualifying it as a hybrid network. Previous studies [15,16,18,19,20,21,25] explored mobile nodes in ring topologies, while others [6,14] have focused on rectangular monitoring regions. Additional approaches in the literature emphasize barrier coverage [5,6]. Although Voronoi diagrams are commonly used for mathematical modeling [3,24], this study uses graph theory as a fundamental framework for network representation.

Regarding determining the minimum quantity of mobile sensor nodes, the proposed method stands out by incorporating scenarios for handling agent failures and malfunctions. It also defines the precise positions required for full coverage of a circular area, assuming a uniform distribution of mobile nodes, thus increasing the robustness and reliability of the monitoring system.

The innovation and originality of this work is a coverage methodology, encompassing models, methods, and algorithms for monitoring hostile environments via a hybrid WSN. The proposal presented in this methodology is oriented toward developing models that provide full coverage and can be applied to the monitoring of aquatic ecosystems and environmental disaster areas. Consequently, this study aims to contribute a methodology to determine the minimum quantity of mobile sensor nodes in a hybrid WSN, which guarantees full coverage without coverage holes and no loss of connectivity among the network’s agents. The mobile nodes are distributed in order to increase the use of the sensors’ detection circumference. In addition, the proposed methodology includes a fault treatment; in the case of sensor node failure, the coverage region is dynamically adjusted, thus guaranteeing full coverage.

The remainder of the paper is organized into five sections. In Section 2, the problem formulation is described, where considerations of the proposed coverage scheme are presented. Section 3 presents the mathematical modeling of the hybrid WSN presented in the previous section. A method to determine the minimum quantity of mobile sensor nodes required for full coverage of the hybrid WSN is presented in Section 4. Section 5 presents the results and discussions, where the data obtained from the computational experiments of the proposed method to determine the minimum number of mobile sensor nodes are analyzed and compared with the Voronoi method. Another analysis is performed regarding variation in the monitoring circumference, in addition to a comparison with the rectangular area. Finally, Section 6 presents the conclusion of the work, highlighting the contributions of the research.

## 2. Problem Formulation

A method to determine the minimum quantity of mobile sensor nodes required in a hybrid WSN is presented in this section, considering a circular monitoring region. The mobile sensor nodes are allocated along a circumference with a radius smaller than that of the monitoring region, whereas the static sensor node is positioned in the center of this circumference.

**Figure 1:**
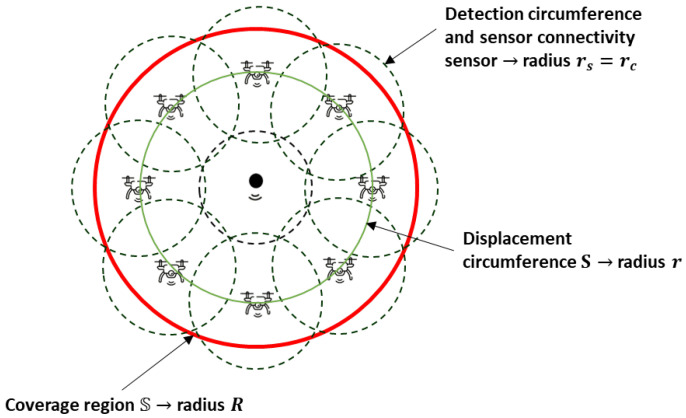
Coverage scheme without holes and with mobile sensor nodes and a static sensor node; the delimitation of the monitoring region is S. The mobile nodes move on circumference *S*, representing the detection and connectivity on circles of radius $r_{s}$ and $r_{c}$, respectively, where $r_{s}=r_{c}$.

Consequently, consider a network with *i* mobile agents, where $i\in I_{N}=\left\{2,\cdots ,N+1\right\}$, the first node is static, and the other *N* nodes are dynamic; then, the hybrid WSN constitutes N+1 sensor nodes. The mobile sensor nodes are allocated in a circle *S* of radius *r*, and the static sensor node is allocated at the center of *S*, as illustrated in Figure 1. The region to be monitored is also a circumference called S of radius *R*. Assume that $q_{i}$ is the angular position of each agent that is counterclockwise measured from the positive horizontal axis. A limitation factor of this sensor network is the detection radius of the sensors $r_{s}$ and the connectivity radius $r_{c}$; this is used to avoid coverage holes caused by an unmonitored region or a lack of communication among the agents. Moreover, consider that all the sensors have the same detection and connectivity radius and that these two radii are equal $r_{s}=r_{c}$.

Each sensor node has information about the angular position of its adjacent neighbors, in which the two neighbors of the *i*-th agent are denoted by

(1) $$\begin{array}{ccc} i^{+} & = & \left\{\begin{array}{ccc} i+1 & \mathrm{if} & i=2,\ldots ,N\\ 2 & \mathrm{if} & i=N+1 \end{array}\right. \end{array}$$

and

(2) $$\begin{array}{ccc} i^{-} & = & \left\{\begin{array}{ccc} N+1 & \mathrm{if} & i=2\\ i-1 & \mathrm{if} & i=3,\ldots ,N \end{array},\right. \end{array}$$

where $i^{+}$ is the right and $i^{-}$ is the left adjacent neighbors of the *i*-th agent, respectively, and *N* is the number of mobile sensor nodes; all sensor nodes have information about the position of the static sensor, located at the center of the circle. The distance between the *i*-th sensor node and its immediate $i^{+}$ neighbor in the counterclockwise direction is defined by $d_{i}\left(k\right)$, and it assumes values in the range [0,2πr) and $\sum_{i=2}^{N+1}d_{i}\left(k\right)=2\pi r$ m.

The hybrid WSN and its modeling are performed by using the $G\left(V,E\right)$ graph, where *V* represents the nodes that are the vertices of the graph, *E* represents the edges [26]; that is, the angular distance between sensor *i* and its adjacent neighbors $\left(i^{-},i^{+}\right)$, with i=2,⋯,N+1. As the sensor node i=1 is a static type that is in the center of the circle, we will not take it into account, as it will not influence the dynamics of the system. Consequently, the graph that describes this relationship is undirected, where V={2,3,…,N,N+1} and $E=\{\left(2,3\right),\left(2,N+1\right),\cdots ,\left(i,i^{+}\right),\left(i,i^{-}\right),\cdots ,\left(N+1,2\right),\left(N+1,N\right)\}$.

The desired position of the dynamic sensor nodes is one that provides flawless coverage—that is, without coverage holes and maintaining network connectivity—for the computed quantity of mobile sensor nodes. The quantity of sensor nodes will depend on the size of the region to be monitored, as well as the detection radius and the connectivity radius of the sensors.

The proposed problem formulation intends to minimize/eliminate coverage holes, which are related to the angular position of the mobile sensor nodes, and maintain the connectivity of the wireless sensor network, avoiding communication failure or even the loss of an agent. Consequently, it is a circular formation problem, which must occur in a timely manner and remain to avoid coverage holes.

## 3. Hybrid WSN Modeling

The proposed hybrid wireless sensor network (WSN) is modeled based on graph theory since interaction between adjacent nodes is necessary for the network to achieve its intended purpose. Then, consider that the dynamic sensor nodes move in a single direction, counterclockwise, with different velocities $\omega_{i}\left(k\right)\neq \omega_{i+1}\left(k\right)\neq \cdots \neq \omega_{N+1}\left(k\right)$ for i=2,⋯,N+1. This modeling takes into account the relative position of neighboring sensor nodes and their angular velocity. The control effort is denoted by $u_{i}$, which represents the angular velocity needed to move the dynamic sensor nodes, $u_{i}\left(k\right)=\omega_{i}\left(k\right)$. Consequently, the angular position of each agent evolves in discrete time [16] according to

(3) $$\begin{array}{ccc} q_{i}\left(k+1\right) & = & q_{i}\left(k\right)+\epsilon u_{i}\left(k\right), \end{array}$$

where ϵ>0 is the step size, $q_{i}\left(k\right)$ is the angular position of the *i*-th sensor node at time instant *k*, and $u_{i}\left(k\right)$ is the control effort (angular velocity) at time instant *k*. Considering that each agent has a different maximum velocity, the control inputs are limited by these velocities, represented by

(4) $$\begin{array}{c} 0\leq u_{i}\left(k\right)\leq \omega_{i}\left(k\right),\forall i\geq 2, \end{array}$$

where $\omega_{i}\left(k\right)$ is the maximum velocity of the *i*-th agent, and this velocity is varied in time *k*. This saturation [25] is applied according to

(5) $$\begin{array}{ccc} u_{i}\left(k\right) & = & \omega_{max(i)}sat\left(u_{i}\left(k\right)\right),i=2,\cdots ,N+1, \end{array}$$

where $sat\left(u_{i}\left(k\right)\right)=sign\left(u_{i}\left(k\right)\right)\min\{1,|u_{i}\left(k\right)\left|\right\}$, and $\omega_{max(i)}$ is the maximum velocity of the *i*-th sensor node; therefore, the control effort $u_{i}$ is limited by the maximum angular velocity of that agent.

Consequently, the control laws for this proposed hybrid WSN, with *N* mobile sensor nodes located on the circumference *S* (taking into account that the *i*-th sensor node determines its angular velocity of displacement from the velocity of its adjacent neighbors), are given by

(6) $$\begin{array}{ccc} u_{2}\left(k\right) & = & \left(\omega_{2}\left(k\right)+\omega_{N+1}\left(k\right)\right)\left(q_{3}\left(k\right)-q_{2}\left(k\right)\right)\\ & & -\left(\omega_{2}\left(k\right)+\omega_{3}\left(k\right)\right)\left(q_{2}\left(k\right)+2\pi r-q_{N+1}\left(k\right)\right), \end{array}$$

(7) $$\begin{array}{c} u_{i}\left(k\right)=\left(\omega_{i}\left(k\right)+\omega_{i^{-}}\left(k\right)\right)\left(q_{i^{+}}\left(k\right)-q_{i}\left(k\right)\right)-\left(\omega_{i}\left(k\right)+\omega_{i^{+}}\left(k\right)\right)\left(q_{i}\left(k\right)-q_{i^{-}}\left(k\right)\right),\\ i=3,\cdots ,N \end{array}$$

and

(8) $$\begin{array}{ccc} u_{N+1}\left(k\right) & = & \left(\omega_{N+1}\left(k\right)+\omega_{N}\left(k\right)\right)\left(q_{2}\left(k\right)-q_{N+1}\left(k\right)+2\pi r\right)\\ & & -\left(\omega_{N+1}\left(k\right)+\omega_{2}\left(k\right)\right)\left(q_{N+1}\left(k\right)-q_{N}\left(k\right)\right). \end{array}$$

According to Equations (6)–(8), $u_{i}\left(k\right)$ is the control effort given in relation to the angular velocity of the agents. Since these sensor nodes are organized in a circle, an adjustment of 2πr is added to the calculation of the distance between the first and last agent on the circumference. These equations are obtained through graph theory and are important for representing the dynamics of the system, according to Equation (3). By arranging Equations (6)–(8), the control effort in matrix form is given by

(9) $$\begin{array}{ccc} u(k) & = & -L(k)q(k)+F(k), \end{array}$$

where *k* is the discrete time instant, $u\left(k\right)={\left[u_{2},\cdots ,u_{N+1}\right]}^{T}\in {ℜ}^{N}$ represents the control effort vector of the dynamic nodes, $q\left(k\right)={\left[q_{2},\cdots ,q_{N+1}\right]}^{T}\in {ℜ}^{N}$ represents the system states, and $L\left(k\right)\in {ℜ}^{N\times N}$ is the Laplacian matrix given by

(10) $$L(k)=[\begin{array}{cccccc} 2\omega_{2}\left(k\right)+\omega_{3}\left(k\right)+\omega_{N+1}\left(k\right) & -\left(\omega_{2}\left(k\right)+\omega_{N+1}\left(k\right)\right) & 0 & \ldots & 0 & -\left(\omega_{2}\left(k\right)+\omega_{3}\left(k\right)\right)\\ \vdots & \vdots & \vdots & \vdots & \vdots & \vdots \\ 0 & \cdots & -\left(\omega_{i}\left(k\right)+\omega_{i^{+}}\left(k\right)\right) & \left(\omega_{i^{-}}\left(k\right)+2\omega_{i}\left(k\right)+\omega_{i^{+}}\left(k\right)\right) & -\left(\omega_{i^{-}}\left(k\right)+\omega_{i}\left(k\right)\right) & \cdots \\ \vdots & \vdots & \vdots & \vdots & \vdots & \vdots \\ -\left(\omega_{N}\left(k\right)+\omega_{N+1}\left(k\right)\right) & \cdots & 0 & \cdots & -(\omega_{2}\left(k\right)+\omega_{N+1}\left(k\right) & \omega_{2}\left(k\right)+\omega_{N}\left(k\right)+2\omega_{N+1}\left(k\right)) \end{array}]$$

Moreover, according to Equations (6)–(8), $F\left(k\right)\in {ℜ}^{N}$ is a vector given by

(11) $$\begin{array}{ccc} F(k) & = & {\left[\begin{array}{ccccc} -\left(\omega_{2}\left(k\right)+\omega_{3}\left(k\right)\right)2\pi r & 0 & \cdots & 0 & \left(\omega_{N+1}\left(k\right)+\omega_{N}\left(k\right)\right)2\pi r \end{array}\right]}^{T}. \end{array}$$

## 4. Methodology

The methodology presented in this paper applies to a known circular region, as the proposed method to determine the minimum quantity of mobile sensor nodes requires knowledge of the monitoring area’s radius and the sensors’ detection radius. Consequently, this approach is not directly suitable for regions with random geometry. However, the proposed hybrid WSN system can be scaled to cover larger areas with arbitrary geometry through the use of multiple sensor sets, ensuring full coverage.

Before performing the circular formation for full coverage, it is essential to determine the number of mobile sensor nodes, which depends on the size of the region to be monitored, the radius of the sensor nodes’ displacement circumference, and the sensor’s detection and connectivity radius. An adequate quantity of mobile sensor nodes can prevent coverage holes, the loss of communication between sensors, and the costs of idle sensors. Consequently, in this study, a method based on the circumference, intersection point, and trigonometric relations is presented to determine the minimum quantity of dynamic sensor nodes, considering that there is already a static sensor node in the center of the monitoring circumference in order to provide full coverage.

To achieve the goal of a minimum quantity of sensor nodes in the WSN to ensure full coverage, a mathematical framework is proposed in this section; in particular, the presented framework is based on assumption, propositions, theorem, and a corollary to determine the order of the mobile sensors to avoid collisions, the minimum quantity of agents, and full connectivity among them.

**Assumption** **1.**  *To avoid collisions along the trajectory, the order of the sensor nodes remains the same as the initial moment (k=0) such that $0\leq q_{2}\left(k\right)<\cdots <q_{i}\left(k\right)<\cdots <q_{N+1}\left(k\right)<2\pi$.*

**Proposition** **1.**  *To avoid coverage holes, the radius of the coverage region S must be $R<3r_{s}$.*

**Proposition** **2.**  *To avoid coverage holes, in relation to the radius of the coverage region S, the distance between the sensor $q_{1}$ and the other mobile sensors is given by $d\left(q_{1}\left(k\right),q_{i}\left(k\right)\right)=\left|q_{i}\left(k\right)-q_{1}\left(k\right)\right|=r$, i=2,⋯,N+1, and the radius of the agents’ displacement circumference must be $r<2r_{s}$. On the other hand, this radius must also respect the constraint $r>R-r_{s}$ so that the detection range of the sensors is sufficient to cover the edge of the monitoring region.*

**Theorem** **1.**  *According to Propositions 1 and 2, and with the intersection point between two circles, considering that it is a uniform formation problem, the quantity of mobile sensor nodes to obtain full coverage is given by*

(12) $$\begin{array}{c} N=\frac{2\pi }{\alpha }, \end{array}$$

*where α is the displacement angle computed from the intersection point between two circles, i.e., it is obtained by solving the system of nonlinear equations.*

**Figure 2:**
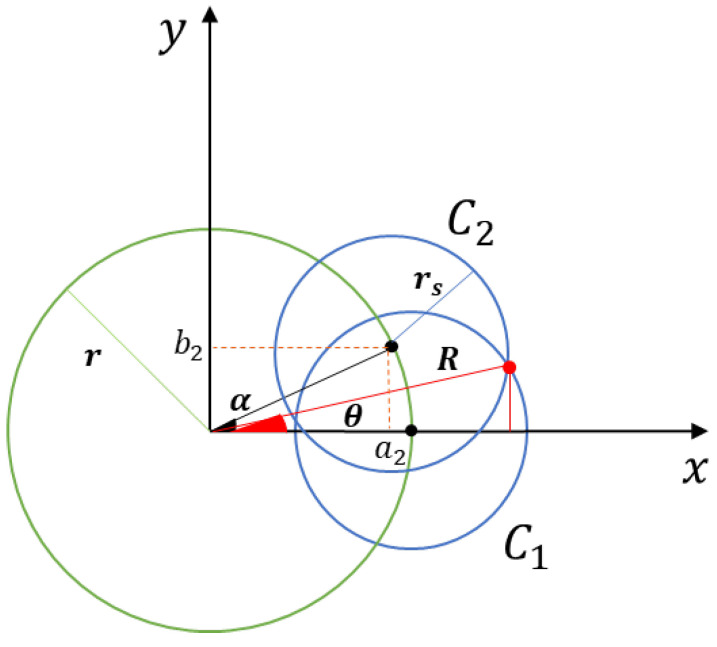
Relationship between the monitoring and detection regions, with the representation of the circles $C_{1}$ and $C_{2}$ in the XY plane and among each other, where *r* is the agents’ displacement radius, $r_{s}$ is the sensors’ detection radius, *R* is the radius of the monitoring, θ is the angle of the intersection point, α is the displacement angle, and $a_{2}$ and $b_{2}$ are the coordinates of the center of $C_{2}$.

**Proof of** **Theorem 1.**  We consider that the center of the displacement circumference *S* of the mobile sensor nodes is the origin of the Cartesian plane XY. Therefore, assuming that the circumference $C_{1}$ is located on the *x* axis with zero displacement angle (α=0), as illustrated in Figure 2, its equation is given by

(13) $$\begin{array}{c} x^{2}-2xr+r^{2}+y^{2}=r_{s}^{2}, \end{array}$$

where $r_{s}$ is the detection radius of the sensor, and *r* is the radius of the agents’ displacement region, determined in accordance with Proposition 2.

The circumference $C_{2}$ is in angular distance *d* from $C_{1}$; this distance is computed in relation to the displacement angle α, where the displacement is the desired one to avoid covering holes, as illustrated in Figure 2.

According to Figure 2, the value of $b_{2}$ is computed using the trigonometric ratio of the sine, from which we obtain that $b_{2}=r\sin\alpha$. As we have the values of the hypotenuse and the opposite side, the value of $a_{2}$ is obtained by applying the Pythagorean theorem, from which we obtain that $a_{2}=\sqrt{r^{2}-{\left(r\sin\alpha \right)}^{2}}=r\cos\alpha$. Consequently, the equation of the circumference $C_{2}$ is given by

(14) $$\begin{array}{c} x^{2}-2rx\cos\alpha +y^{2}-2yr\sin\alpha +r^{2}=r_{s}^{2}. \end{array}$$

We know that the radius of the monitoring circumference *R* in relation to the intersection point *x* and according to the trigonometric relation is given by $R=\frac{x}{\cos\theta }$. Therefore, we subtract the two equations of the circles and substitute this value of *x* in the equation of circumference $C_{1}$, Equation (13), to obtain the value of *y*, which is the largest coordinate of the *Y* axis, where the two circles intersect. Consequently, just substitute all the known variables in the equation of circumference $C_{2}$, Equation (14), and equalize this *x* found in the equation Rcosθ, where θ is the angle of the intersection point between the two circumferences. Finally, we find the value of the displacement angle α according to the distribution of mobile sensors in the circumference *S*, which is a uniform circle formation problem, and according to Theorem 1, we propose $\alpha =\frac{2\pi }{N}$, where *N* is the number of agents needed to achieve full coverage.

From the inverse of Theorem 1 and Propositions 1 and 2, it is possible to determine the size of the radius of the monitoring circumference (S), which provides full coverage, from a network with *N* dynamic sensor nodes moving in the region *S*. This is based on the solution to the system of equations for the two circumferences under analysis, which is given by

(15) $$\begin{array}{c} \left\{\begin{array}{c} x^{2}-2xr+r^{2}+y^{2}=r_{s}^{2}\\ x^{2}-2xr\sqrt{1-\sin\alpha^{2}}+r^{2}+y^{2}-2yr\sin\alpha =r_{s}^{2} \end{array},\right. \end{array}$$

where *x* and *y* are the intersection point(s) of the circles. Consequently, the radius of the monitoring region *R* is the intersection point *x* furthest from the origin of the Cartesian plane.

**Figure 3:**
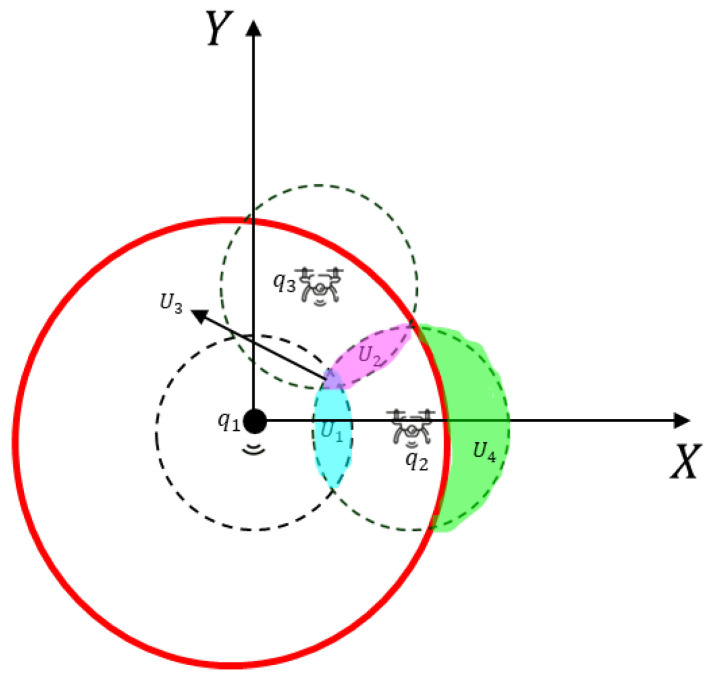
Areas of intersections among the detection circles of the sensor node, between the circumference of the sensor node and the circular monitoring area, in the Cartesian plane.

With the presence of these *N* mobile agents and the static sensor node, full coverage in the proposed network topology is guaranteed if the resulting detection area of the sensor nodes is greater than or equal to the area of the circular region of interest. The resulting detection area, taking into account the intersection areas and according to Figure 3, is given by

(16) $$\begin{array}{c} A_{res}=\left(N+1\right)A_{det}-NU_{1}-NU_{2}-NU_{4}+NU_{3}, \end{array}$$

where $A_{det}=\pi r_{s}^{2}$ is the area of the sensor’s detection circle, *N* is the number of mobile sensor nodes, $U_{1}$ is the intersection area between the detection circle of the static sensor node and of a mobile sensor node, $U_{2}$ is the intersection area between two detection circles of mobile agents, $U_{4}$ is the area of the detection circle of a mobile node that is outside the circular monitoring region, and $U_{3}$ is the intersection area between three detection circles, as shown in Figure 3. Consequently, to ensure full coverage, it is necessary that

(17) $$\begin{array}{c} A_{mon}\leq A_{res}, \end{array}$$

where $A_{mon}=\pi R^{2}$ is the total area of the monitoring circumference.    □

**Corollary** **1.**  *For the full connectivity of the network, Theorem 1 ensures full coverage of the circular monitoring region, and assuming $r_{s}=r_{c}$, the connectivity between sensor nodes is also guaranteed.*

According to Corollary 1, network connectivity is ensured by Theorem 1, which determines the full coverage, and considering that the connectivity radius $r_{c}$ is equal to the sensor detection radius $r_{s}$. Consequently, the coverage condition directly implies network connectivity, as the maximum distance allowed among the sensors to guarantee detection also guarantees communication.

The sensor nodes are susceptible to failure due to environmental conditions and power depletion, for example. Therefore, if a sensor node does not send or does not receive its positioning information at time instant *k*, it is assumed that this node has failed. Consequently, this node must be replaced, or the monitoring area must be reduced in order to provide full coverage without holes and with a full connection using the number of sensor nodes available.

According to the converse of Theorem 1, if the quantity of mobile sensor nodes is known, then it is possible to determine the radius *R* of the circular monitoring region that provides full coverage.

Consequently, the above is demonstrated using the equations of the circles $C_{1}$, Equation (13), and $C_{2}$, Equation (14). For this situation, the displacement angle α is computed according to Equation (12). Then, from the solution of the system of equations of the two circles under analysis, Equation (15), the coordinates XY corresponding to the intersection point between these two circles are obtained.

From the coordinates XY of the intersection point between circles $C_{1}$ and $C_{2}$, the angle θ of this intersection point is calculated, as illustrated in Figure 2, which is given by

(18) $$\begin{array}{c} \theta =\tan^{-1}\frac{y}{x}+\frac{\pi }{2}sign\left(y\right)\left(1-sign(x)\right), \end{array}$$

where θ is the angle of the intersection point of circles $C_{1}$ and $C_{2}$, $\tan^{-1}\left(.\right)$ is the inverse tangent function, and sign(.) is the sign function that returns the sign of the variable, indicating 1 if it is positive, −1 if it is negative, and 0 if it is zero. The result of this equation is the angle in radians between the positive axis *X* and the intersection point given by the coordinates *X* and *Y*.

Consequently, according to Figure 2, the radius of the monitoring circle *R* (knowing the angle and the coordinates of the intersection point, taking into account the trigonometric relation) is given by

(19) $$\begin{array}{c} R=\frac{x}{\cos\theta }, \end{array}$$

where *x* is the coordinate of the *X* axis of the intersection point, and θ is the angle of this intersection between the two circles.

## 5. Results

In this section, we detail the application of the proposed methodology to determine the minimum quantity of dynamic sensor nodes required for a hybrid WSN, providing full coverage and avoiding loss of network connectivity and coverage holes. The results obtained are based on the formulation presented in Section 2, where the equations from this section are applied in the development of Algorithm 1. The computational experiments results are obtained with MATLAB R2023a software.

According to Algorithm 1, the first step is to define the values of the known parameters, which are the following: the radius of the circumference to be monitored (R), the radius of the sensor detection circumference that is considered equal to the radius of the sensor connectivity circle $\left(r_{s}=r_{c}\right)$, and the radius of the agents’ displacement circle (r), which is defined according to Proposition 2; this creates the symbolic variable *N*, which is defined as a function of α, according to Equation (12).

The second step is to compute the equations of two circumferences. To facilitate the computations, the circle $C_{1}$ is in the angular position 0 rad (zero radians); that is, in relation to the Cartesian plane xy, and its center is displaced by $x_{c_{1}}=r$, which is the radius of the displacement circumference, and $y_{c_{1}}=0$. The second circle $C_{2}$ is in the angular position α rad, as shown in Figure 2. Consequently, in relation to the Cartesian plane xy, the coordinates of its center are defined based on trigonometry and the Pythagorean theorem, from which we obtain that $x_{c_{2}}=\sqrt{r^{2}-{\left(r\sin\alpha^{2}\right)}^{2}}$ and $y_{c_{2}}=r\sin\alpha$. Therefore, we have a set of equations, with the coordinates of the intersection points $\left(X_{c}\left(\alpha \right),Y_{c}\left(\alpha \right)\right)$ between the circumferences $C_{1}$ and $C_{2}$ given as a function of α.
**Algorithm 1** Defining the Minimum Quantity of Sensor Nodes▹ **Setup—Parameter Initialization**1:R← monitoring region radius value;2:$r_{s}\leftarrow$ sensor detection radius value;3:r← value of the radius of the displacement circumference;4:$\alpha =\frac{2\pi }{N}-N\leftarrow$ wanted value from sensor nodes;▹ **Intersection Point Computation**    **Equation of the Circumference 1**5:$C_{1}\leftarrow x^{2}-2a_{1}+a_{1}^{2}+y^{2}-2b_{1}+b_{1}^{2}-r_{1}^{2}$;6:$a_{1}\leftarrow r$;7:$b_{1}\leftarrow 0$;8:$r_{1}\leftarrow r_{s}$;    **Equation of the Circumference 2**9:$C_{2}\leftarrow x^{2}-2a_{2}+a_{2}^{2}+y^{2}-2b_{2}+b_{2}^{2}-r_{2}^{2}$;10:$a_{2}\leftarrow \sqrt{r^{2}-{\left(r\sin\alpha^{2}\right)}^{2}}$;11:$b_{2}\leftarrow r\sin\alpha$;12:$r_{2}\leftarrow r_{s}$;  **Solve system of equations**13:$\left\{\begin{array}{c} X_{\max_{c}}\left(N\right)\;Y_{\max_{c}}\left(N\right) \end{array}\right.\leftarrow \left\{\begin{array}{c} X_{c}\left(\alpha \right)\;Y_{c}\left(\alpha \right) \end{array}\right.\leftarrow \left\{\begin{array}{c} C_{1}\;C_{2} \end{array}\right.$;    **Computation of the intersection angle**14:$\theta \left(N\right)\leftarrow \tan^{-1}\frac{Y_{\max_{c}}\left(N\right)}{X_{\max_{c}}\left(N\right)}+\frac{\pi }{2}sign\left(Y_{\max_{c}}\left(N\right)\right)\left(1-sign(X_{\max_{c}}\left(N\right)\right);$  **Computation of equations in relation to angular position**15:$\left\{\begin{array}{c} E_{1}\left(N\right)\leftarrow X_{\max_{c}}\left(N\right)-R\cos\theta \left(N\right)\;E_{2}\left(N\right)\leftarrow Y_{\max_{c}}\left(N\right)-R\sin\theta \left(N\right) \end{array}\right.$  **Solution of the system of nonlinear equations**16:$N\leftarrow \left\{\begin{array}{c} E_{1}\left(N\right)\;E_{2}\left(N\right) \end{array}\right..$

The third step is to compute the angle of this intersection point (θ), but this angle is computed only for the largest value obtained in $X_{c}\left(\alpha \right)$ and the corresponding value of $Y_{c}\left(\alpha \right)$. This value of θ is already given as a function of *N*, according to the relationship between *N* and α, Equation (12), as it is the desired variable, which is the minimum quantity of sensor nodes with which to monitor a circular region without failures. Next, the new equations are computed by equating the value of the coordinates $\left(X_{\max_{c}}\left(N\right),Y_{\max_{c}}\left(N\right)\right)$ to the values of the coordinates computed from the intersection angle θ(N) based on the trigonometric functions. This results in a new system of nonlinear equations $\left(E_{1}\left(N\right),E_{2}\left(N\right)\right)$. Finally, to obtain the value of *N*, it is sufficient to solve the system of nonlinear equations; with this quantity of mobile sensor nodes, the desired angular positions of the sensor nodes (α) are determined.

To monitor the coverage region, a circumference of R=10 m radius is required, where the sensor detection radius is equal to the connectivity radius $r_{s}=r_{c}=6$ m, and according to Proposition 2, the radius of the displacement circumference is r=7 m. By applying Theorem 1, the quantity of mobile sensor nodes is five, considering that there is a static sensor node at the center of the circle; therefore, in total, the hybrid WSN has a construction of six sensor nodes.

**Figure 4:**
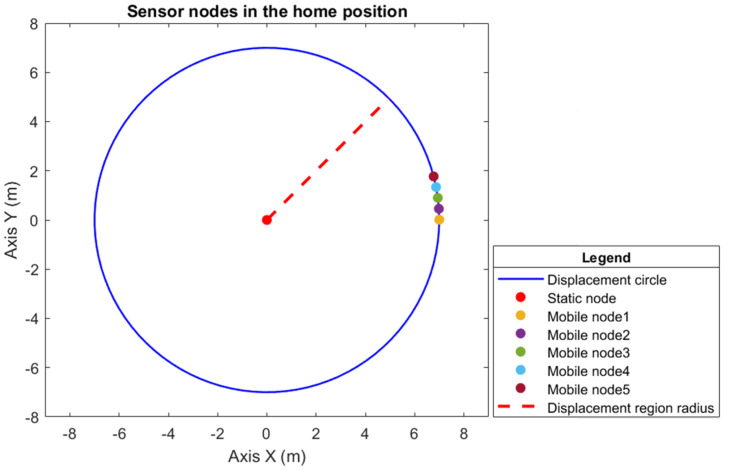
The initial positions of the six sensor nodes, where the mobile sensor nodes are allocated along the circumference of area *S* with a radius of r=7 m.

**Figure 5:**
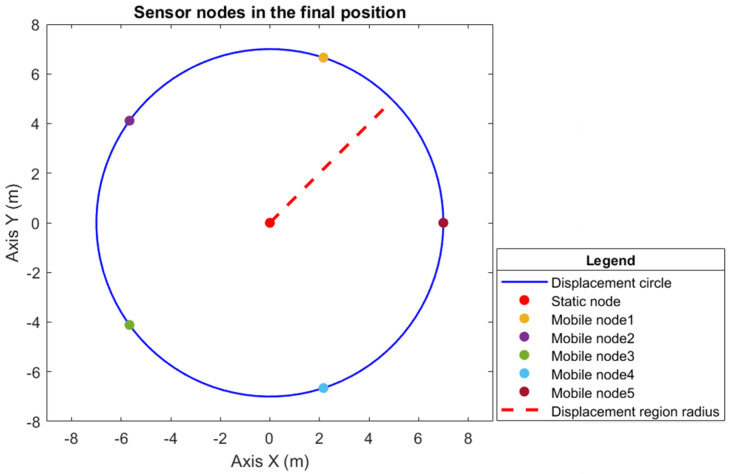
Desired position of the six sensor nodes in the displacement circumference *S*, with radius r=7 m, where the five agents are uniformly distributed in *S*, and the static sensor node is located in the center.

The agents are labeled counterclockwise according to their initial positions on the circumference, and each agent has a maximum speed. The initial position of each agent is given by ${\left[\begin{array}{cccccc} 0.0143 & 0.5343 & 1.0543 & 1.5743 & 2.0943 & 0 \end{array}\right]}^{T}$ rad, considering the last position to be that of the static sensor node, which is at the center of the circumference. For this position, since there is no defined angular position in relation to the circle, an arbitrary convention is made where this position is determined by 0 rad (zero radians). The maximum angular velocity of the sensor nodes is ${\left[\begin{array}{cccccc} 1.2 & 1.9 & 2.6 & 3.3 & 4 & 0 \end{array}\right]}^{T}$ rad/s, considering that the last speed is that of the static node; therefore, it is 0 rad/s (zero radians). Figure 4 shows the sensor nodes in their initial positions, and Figure 5 shows the sensor nodes in their final position, which is the desired position (i.e., evenly distributed around the displacement circumference).

Figure 4 shows the sensor nodes in their initial positions on the displacement circumference, and Figure 5 shows the sensor nodes in their final position, i.e., the desired position, uniformly distributed on the displacement circumference. Therefore, the positions specified to provide full coverage of the circular region of radius R=10 m are ${\left[\begin{array}{cccccc} 8.79 & 17.59 & 26.39 & 35.18 & 43.98 & 0 \end{array}\right]}^{T}$ m, which, when transformed into angular position, gives ${\left[\begin{array}{cccccc} 1.25 & 2.51 & 3.77 & 5.02 & 6.28 & 0 \end{array}\right]}^{T}$ rad.

**Figure 6:**
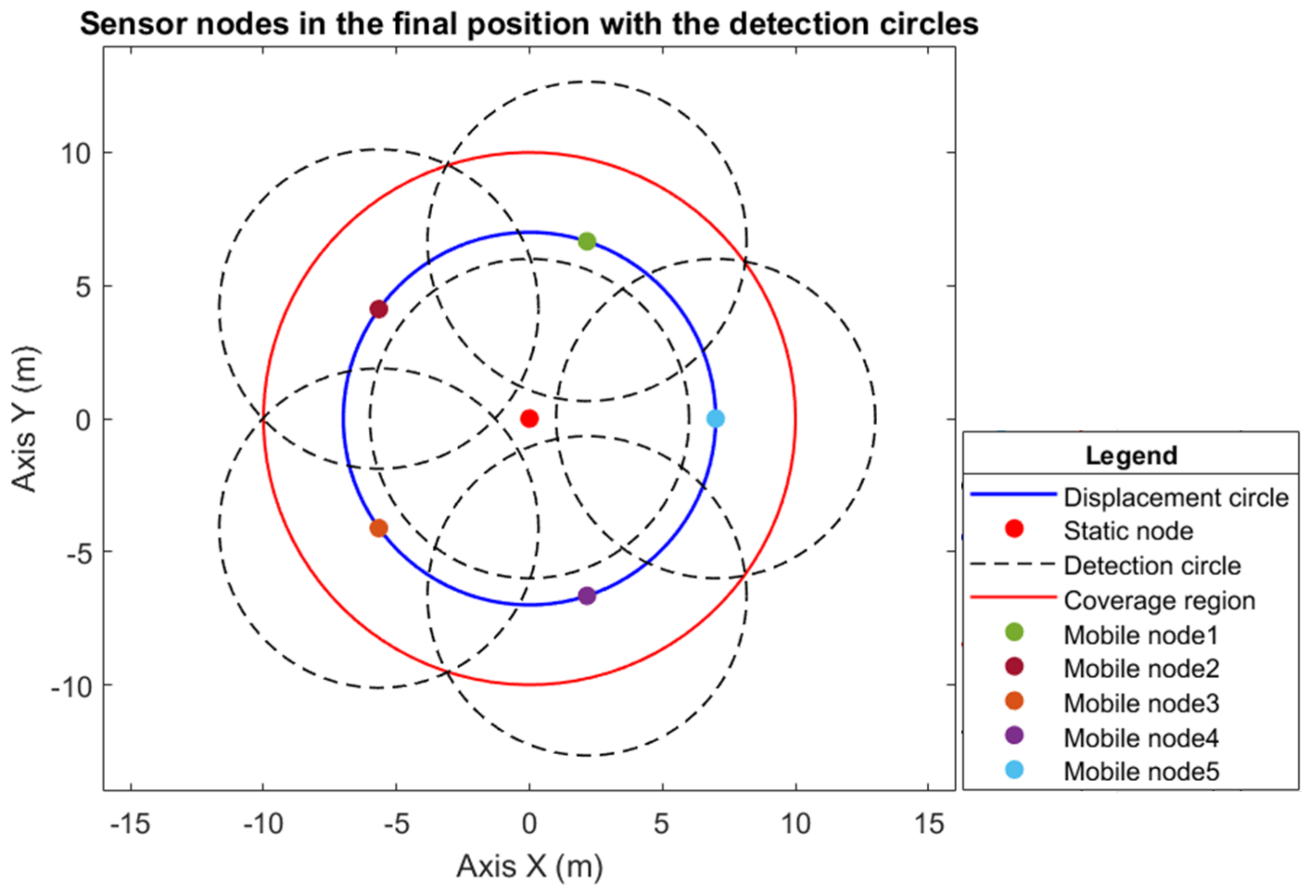
Full coverage of a circular region S, with radius R=10 m, consisting of six sensor nodes, with five dynamic nodes allocated in the displacement circle and 1 static node in the center. The detection circles of the sensors are also presented.

Theorem 1 guarantees that the quantity of mobile sensor nodes determined is the minimum that is sufficient to provide full coverage without holes and without loss of connectivity among the nodes. Figure 6 illustrates the monitoring region S, the displacement circumference of the sensor nodes, and the computed quantity of agents in their desired positions. In addition to the illustration of the detection and connectivity circumference (which are considered to be equal to $r_{c}=r_{s}$), the efficiency of the theorem in computing the necessary quantity of mobile sensor nodes, together with the hybrid WSN topology used, is proven, as it provides full coverage.

**Figure 7:**
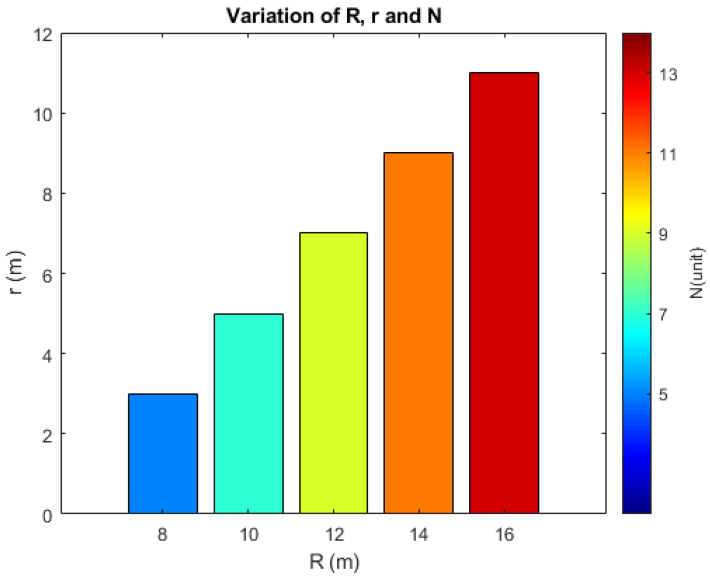
Bar graph showing the variation in the radius *R* of the circular monitoring region and the values obtained for the radius *r* of the displacement circumference, as well as the minimum quantity *N* of mobile sensor nodes needed for full coverage.

To evaluate the efficiency of the proposed methodology in determining the minimum quantity of mobile sensor nodes required to ensure full coverage of the circular monitoring region, the radius *R* of this monitoring region was varied, as established in Proposition 1. Consequently, the radius *r* of the displacement circumference also varies, according to Proposition 2. In this analysis, the detection and connectivity radius of the sensors are considered constant, adopting $r_{s}=r_{c}=6$ m. Figure 7 presents the results obtained for these variations.

According to Figure 7, the radius *R* of the monitoring region varied from 8 to 16 m, according to the condition $R<3r_{s}$. Due to this variation, the radius *r* of the agents’ displacement circumference varied from 3 to 11 *m*, respecting the interval $R-r_{S}<r<2r_{s}$. When analyzing the results obtained for the minimum quantity of mobile sensor nodes, *N*, a directly proportional relationship is observed: as the values of *R* and *r* increase, the minimum quantity of mobile sensor nodes that guarantees complete coverage of the circular region also increases.

**Figure 8:**
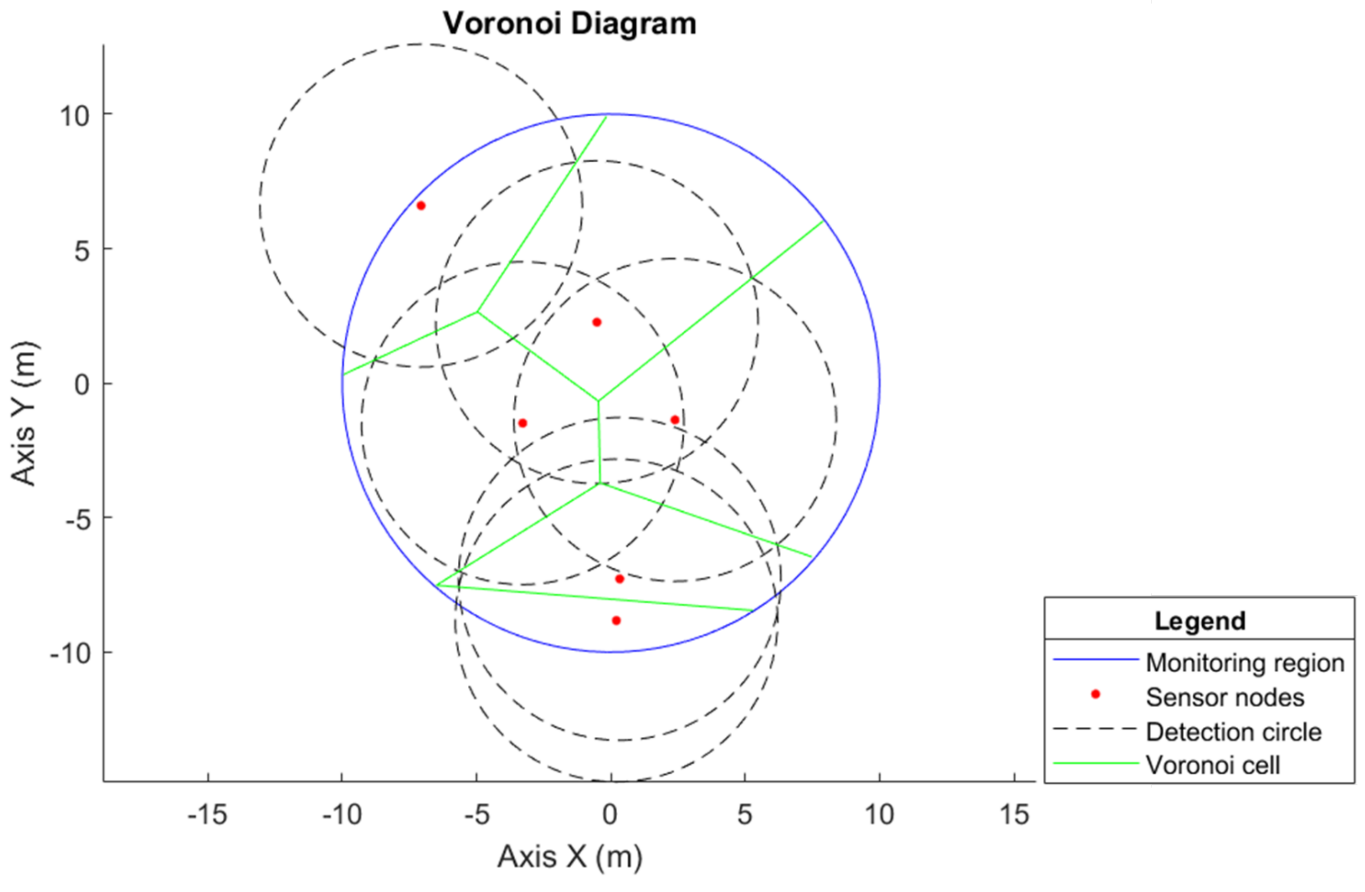
Voronoi diagram for a circular monitoring region of radius R=10 m, consisting of six agents allocated based on the Voronoi cell; the detection circles of the sensors are also illustrated.

The Voronoi diagram can be applied to any geometry, including the circular region [27]. The Voronoi diagram is used to compare the proposed methodology, as it is a widely used method regarding network coverage. Consequently, to allocate the six sensor nodes in the monitoring circumference with a radius of R=10 m, considering the sensor nodes at arbitrary points along the circumference, the obtained Voronoi cell is shown in Figure 8.

When comparing the Voronoi method for network coverage with the proposed model, as in Figure 6, it is observed that it is still necessary to improve the positioning of the sensor nodes since, in the configuration presented in Figure 8, there are still many coverage holes.

In the proposed hybrid WSN model, when monitoring a circular region with a radius of R=10 m, it is guaranteed that the six determined sensor nodes represent the minimum number necessary to perform monitoring without loss of connectivity and without having coverage holes. This is in addition to also obtaining the desired position of the agents since it is known that they are distributed uniformly. For this Voronoi model, it is necessary to compute the desired position of the sensor nodes and analyze network connectivity to see whether full coverage is provided.

**Figure 9:**
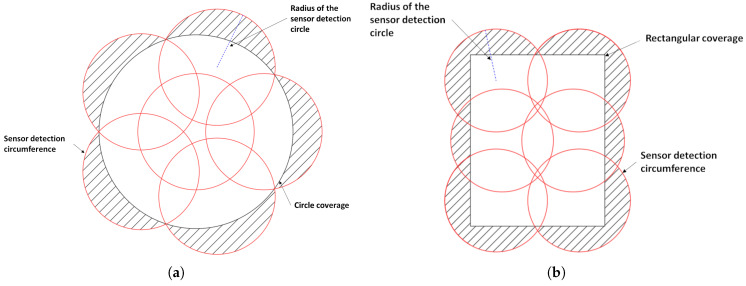
Monitoring models: (**a**) proposed circular model; (**b**) rectangular model with the same area and the same quantity of sensor nodes.

When comparing a circular monitoring region with a rectangular one (both with the same area), it is observed that the use of the sensors’ detection area is greater in the circular region, although this configuration has a larger overlap area between the sensors. This overlap, however, increases the reliability of the network since more than one sensor covers the same area, providing redundancy. Considering a circular monitoring region with a radius of R=10 m, the total area is 314.16 $m^{2}$. According to Theorem 1, the minimum quantity of dynamic sensor nodes required to ensure full coverage is five, totaling six sensor nodes when including the static sensor. In this coverage scheme, the unused detection area is $A_{usel}=141.29$ $m^{2}$, as illustrated in Figure 9a.

A rectangular monitoring region of the same area (314.16 $m^{2}$), where the width is $W_{d}=20$ m and the length is $L_{g}=15.708$ m, also requires six sensor nodes to ensure full coverage. However, the unused sensing area is $A_{usel}=195.84$ $m^{2}$, as shown in Figure 9b. Consequently, it is concluded that the circular region provides better usage of the sensing area regarding the sensor nodes and ensures greater reliability for the network. This is because a larger portion of the area is covered by more than one sensor. This demonstrates that the circular geometry is more efficient in terms of coverage, and the rectangular configuration presents a significantly larger unused area.

**Figure 10:**
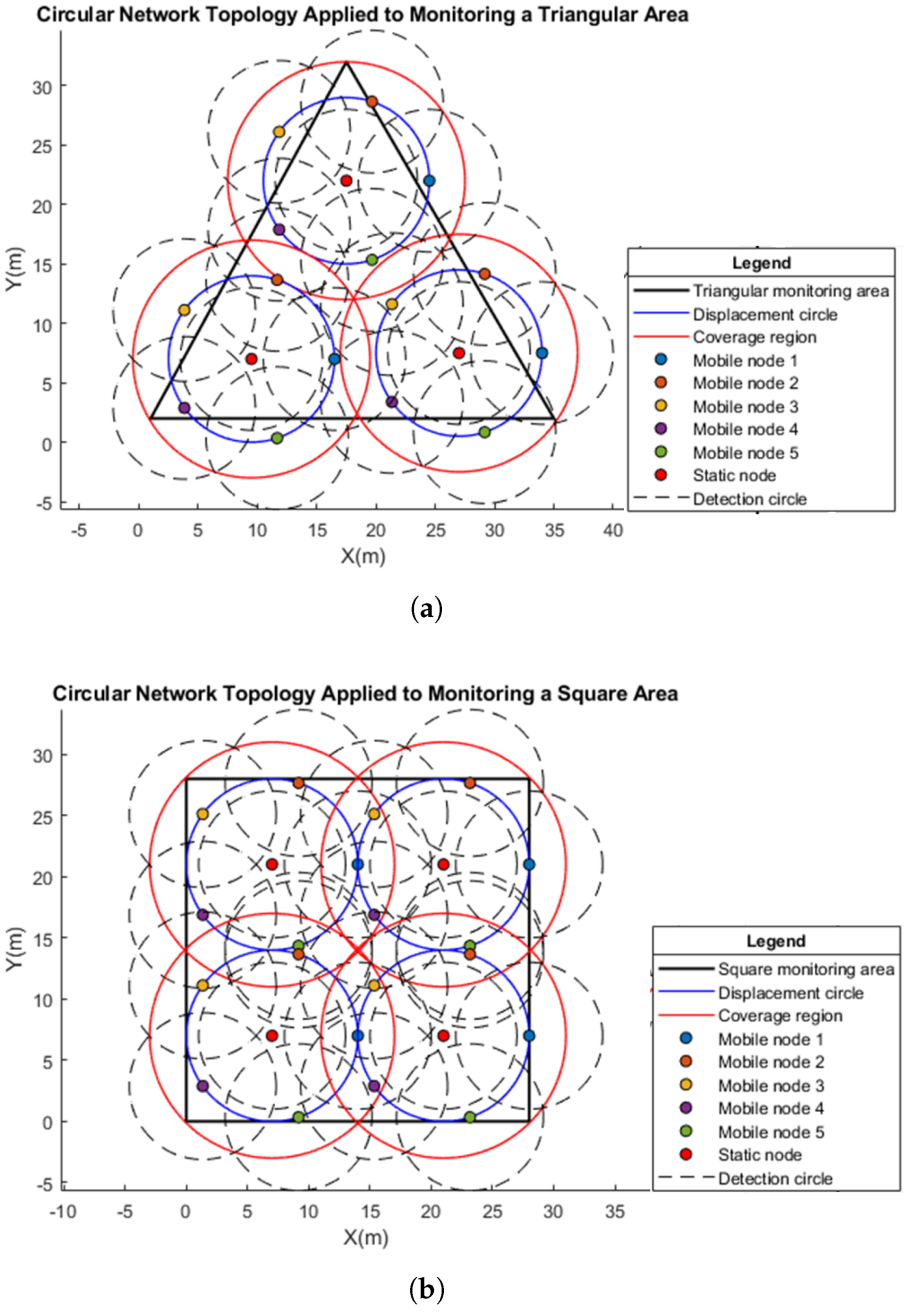
Different monitoring areas using the proposed circular topology: (**a**) triangular region monitored by three circular networks; (**b**) square region monitored by four circular networks.

This proposed circular network topology is capable of monitoring regions with any geometric shape. To do this, it is sufficient to organize multiple circular networks together, covering the entire area of interest, as illustrated in Figure 10. In this study, we chose a circular monitoring area, as it allows better use of the detection region of the sensor nodes, as shown in Figure 9.

Figure 10 demonstrates that the proposed circular network topology is versatile and can be applied to monitoring regions with different geometric shapes. In Figure 10a, a triangular area is completely covered by three circular networks. In Figure 10b, the coverage of the square region is carried out by four networks with the same circular topology, ensuring full monitoring of the area.

**Figure 11:**
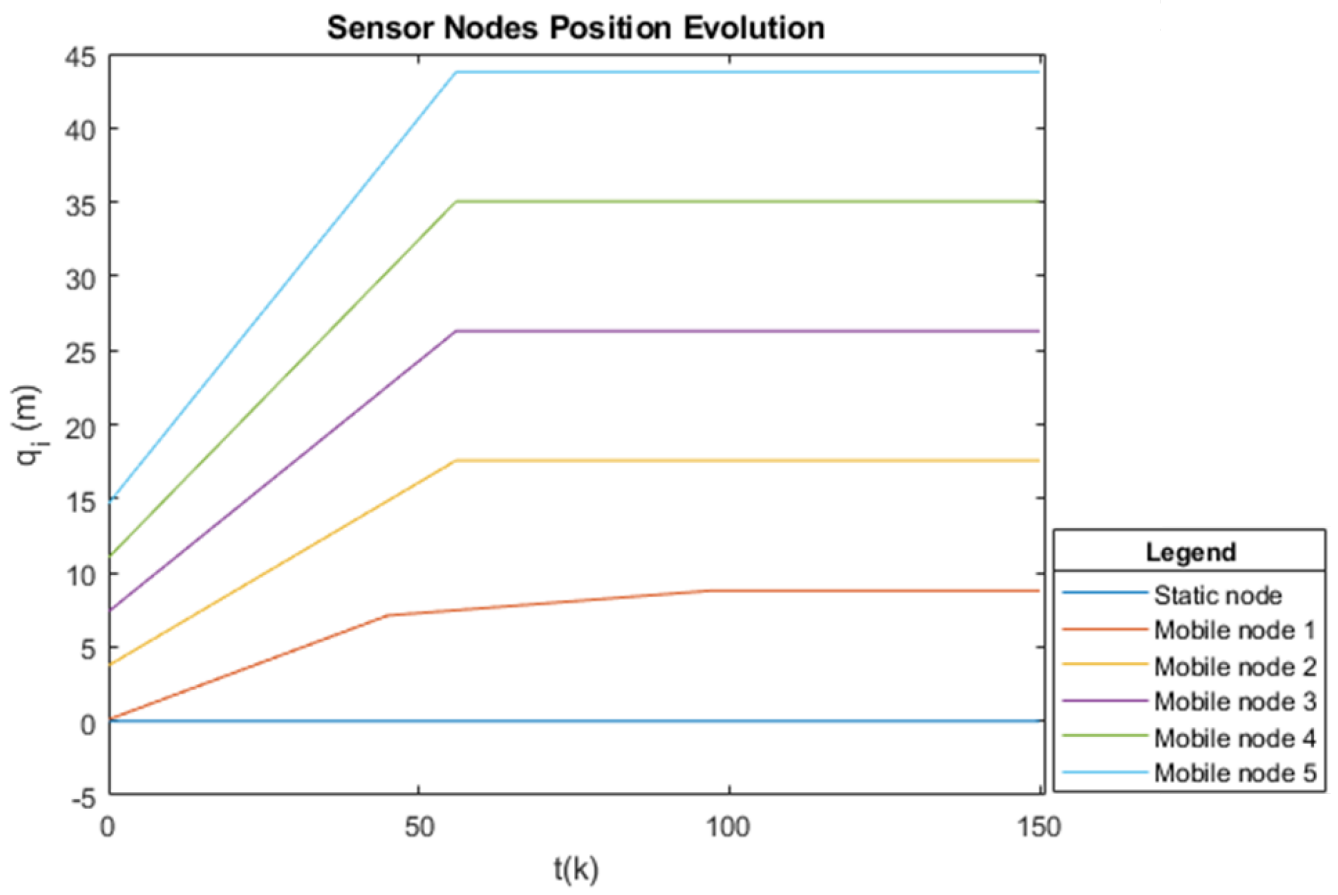
Collision-free displacement of the five agents in time *k* to reach the desired position, with the representation of the permanence of the static sensor in the center of the circumference.

The displacement of the agents was driven by closed-loop control, in which each node computes the control effort required to perform displacement in relation to the final desired position, according to Equation (5). Since the control effort is considered to be the angular velocity, this new velocity is used to update the Laplacian matrix regarding angular velocity at time instant *k*. This displacement along the circumference of the radius *r*, from the initial position to the desired point, is illustrated (in meters) in Figure 11. It is observed that it is a linear displacement, and it is confirmed that the sensor nodes maintain the initial order (i.e., no agent overtakes another), thus avoiding collision during the construction of the hybrid WSN topology.

**Figure 12:**
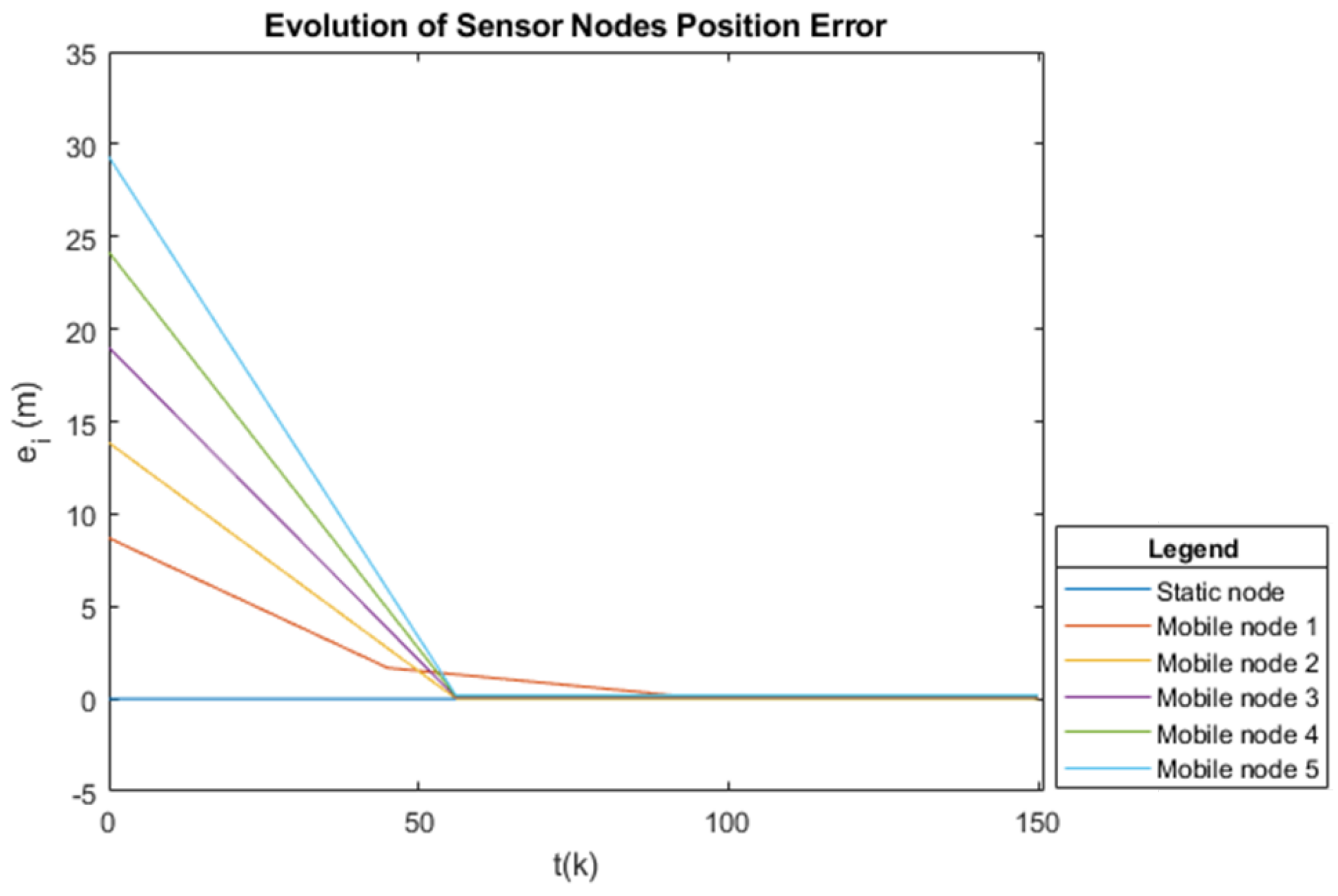
Error in the desired angular position at time *k* for the six sensor nodes, where all tend to a zero error.

Figure 12 illustrates the error of the angular position of the agents, also given in meters, in which it is observed that the error decreases as the agents move to the final position. After approximately k=60 time instants, most of the sensor nodes have already reached the desired position, and their errors tend to zero. However, mobile sensor node 1 takes longer to reach its desired final position, particularly because its maximum speed is the lowest. The error of the static sensor node will always be zero, as it is already at its desired location and does not need to move.

Consequently, the proposed method was validated through simulations, as demonstrated in Figure 6, Figure 9, Figure 11 and Figure 12. These simulations show that the system behaves as expected, providing full coverage of the circular monitoring region and, thus, validating the proposed methodology.

A real-world application of this hybrid WSN is the monitoring of offshore oil platforms, representing a difficult environment to access where external factors, such as wind and ocean currents, cause unpredictable node movement. In this context, the proposed hybrid network with mobile sensor nodes provides the network with greater flexibility and autonomy to overcome these disturbances. Communication among agents can be performed via LoRa due to its long range, which is ideal for applications in hostile areas. In addition, it has low energy consumption, which contributes to extending the agents’ useful life, especially considering the fact that drones have limited flight time due to energy consumption.

## 6. Conclusions

Aiming at the dynamic monitoring of a hybrid WSN without coverage holes and loss of sensor node connectivity, a method for computing the minimum quantity of mobile sensor nodes to fully monitor a given circular region was presented in this paper. Next, mathematical modeling of sensor node allocation in this network was performed based on graph theory, taking into account position and angular velocity.

The proposed method for determining the number of agents was demonstrated to be efficient in forming a hybrid WSN with a static sensor node in the center of the circle. This has the advantage of avoiding the expense of an idle sensor node in addition to the fact that, when mathematical modeling is applied and the mesh is closed, full coverage is achieved without loss of connectivity, coverage failure, or collision. This is because the model guarantees that the mobile sensor nodes move while preserving the initial order. It also guarantees the agents’ desired angular position, as it considers that they are evenly distributed along the displacement circumference.

Another advantage of this model is its better use of sensor detection areas, as the circular arrangement provides a greater intersection in the coverage zone. This, in turn, increases the redundancy and reliability of the network, ensuring more accurate detection of events or anomalies in the environment.

A disadvantage of this method is the system of nonlinear solutions needed to obtain the appropriate number of agents. This process can be computationally complex and requires more time and solution convergence to ensure that an efficient solution is obtained.

Consequently, the hybrid WSN model and the method for determining the number of agents correspond to the desired results, showing that it can be used to monitor hostile environments, difficult-to-access environments, or any region of interest. In this case, sensor nodes capture information that can be processed by each node or at a base station for specific decision-making.

## Data Availability

The data presented in this study are available upon request from the corresponding author.

## Abbreviations

The following abbreviations are used in this manuscript:

WSN	Wireless sensor network
QoS	Quality of service
